# BODY COMPOSITION AND ANTHROPOMETRICS OF OLDER ADULTS AT DIFFERING LEVELS OF URBANIZATION IN CHINA

**DOI:** 10.1093/geroni/igae098.2753

**Published:** 2024-12-31

**Authors:** Hillary Spangler, Lisa Michelson, Annie Green Howard, Hsaio Tien, David Lynch, Shufa Du, Penny Gordon-Larsen, John Batsis

**Affiliations:** University of North Carolina School of Medicine, Chapel Hill, North Carolina, United States; University of North Carolina School of Medicine, Chapel Hill, North Carolina, United States; University of North Carolina, Chapel Hill, North Carolina, United States; University of North Carolina, Chapel Hill, North Carolina, United States; University of North Carolina School of Medicine, Chapel Hill, North Carolina, United States; University of North Carolina, Chapel Hill, North Carolina, United States; University of North Carolina, Chapel Hill, North Carolina, United States; University of North Carolina, Chapel Hill, North Carolina, United States

## Abstract

**Background:**

Less urbanized areas in China are associated with higher risk of developing frailty. We aim to identify differences in the relationship between body composition and urbanization.

**Methods:**

Using the China Health and Nutrition Survey data in 2015, we included community-dwelling adults aged ≥60 with bioelectrical impedance data assessing fat mass and fat-free mass (body composition). Urbanization was based on a multi-component index (0-120 points) based on 12 community-level variables, with lower scores indicating lower urbanization. Latent class analysis was used to identify underlying heterogeneous patterns of body composition classes. Continuous and categorical variables were compared using Chi-squared and ANOVA tests, respectively.

**Results:**

Of 1,424 participants, 55% were female, mean age 72.0±5.9 and BMI 23.8±3.6. Three of the five best fitting classes aligned with robust (n=517, moderate BMI, body fat, muscle), pre-frail (n=602, low BMI, low body fat, low muscle), and frail (n=211, lowest BMI, % body fat, and muscle) statuses. Urbanization index was significantly different between the five classes (p< 0.001). Urbanization index was lowest (65.1) for class that aligned with frailty; mean age 72.0 ±5.9, 54.7% female. The class aligning with robust status was associated with higher urbanization index (71.6); mean age 71.9 ±5.7, 54.6% female.

**Discussion:**

Older adults with body composition consistent with frailty had a lower level of urbanization. Urbanization level may influence body composition in China.

